# CEO cultural background and overinvestment decisions: The north-south divide in China

**DOI:** 10.1371/journal.pone.0288703

**Published:** 2023-11-15

**Authors:** Chia-Hsien Tang, Yen-Hsien Lee, Ya-Ling Huang, Wen-Ting Chang

**Affiliations:** 1 College of Accounting and Auditing, Guangxi University of Finance and Economics, Guangxi Accounting Research Institution (Research Institute for Applied Accounting Measurement Methods), Nanning, Guangxi, China; 2 College of Business, Department of Finance, Chung Yuan Christian University, Taoyuan City, Taiwan; 3 Department of Golden-Ager Industry Management, Chaoyang University of Technology, Taichung, Taiwan; Yunnan Technology and Business University, CHINA

## Abstract

This study addresses an under-researched area in corporate behavior by examining the impact of a CEO’s cultural background on corporate overinvestment decisions. We focus on the unique cultural dichotomy between northern and southern China as our context of study. Additionally, we scrutinize the interactions between a CEO’s age and the type of company ownership in influencing overinvestment tendencies. Our aim is to enrich theoretical understanding of factors influencing corporate overinvestment, offering practical implications for businesses within and beyond China. By filling this gap in the literature, our study sheds light on the nuanced determinants of overinvestment decisions, aiding businesses in refining their investment strategies and governance mechanisms.

## Introduction

Understanding the influence of a CEO’s cultural background on overinvestment decisions is essential, especially in the global economy’s diverse cultural contexts. China provides a particularly interesting case due to its cultural heterogeneity and the significant north-south cultural divide. In listed companies, the separation of ownership and management is a key feature. CEOs, appointed by shareholders, wield significant decision-making power, influencing the company’s operations and investment performance. Although previous research has examined the impact of CEOs’ personal traits on a company’s investment performance [1–3], there has been less focus on the influence of a CEO’s cultural background on overinvestment decisions.

CEO characteristics, such as cultural background, have been found to systematically vary investment behaviors [4]. Moreover, research has indicated that executives’ cultural backgrounds can influence company investment decisions [5]. Despite these findings, the relationship between informal institutions, like a CEO’s cultural background, and investment decisions has been less explored. While existing literature provides a comprehensive understanding of the dominance of state-owned versus private enterprises in the capital market [6], the impact of a CEO’s cultural background on overinvestment decisions remains an under-explored territory. This study aims to address this research gap, providing an empirical investigation into the influence of a CEO’s cultural background on overinvestment, particularly within the unique context of Chinese businesses. Our in-depth examination of the regional cultural differences within China, specifically between the North and South, lends a distinct characteristic to this study.

In this study, we aim to address this research gap by investigating the impact of a CEO’s cultural background on overinvestment in Chinese firms. Using data from listed firms between 2000 and 2018, we examine the influence of cultural background on corporate decision-making and its implications for firm value and investor wealth. In addition to cultural background, we also consider the role of the CEO’s age on overinvestment decisions, based on evidence that younger CEOs tend to take more risks than older ones [7, 8]. We also account for the agency problem and governance issues in state-owned enterprises, which can contribute to overinvestment [6, 9].

This study offers a unique contribution to existing literature by providing an in-depth analysis of the impact of a CEO’s cultural background on overinvestment decisions, a topic not sufficiently explored in previous studies. We focus on the regional cultural differences within China and their influence on overinvestment, adding a unique element to our study. By examining the complex interplay between CEO age, company ownership, and overinvestment behaviors, we hope to provide valuable insights that extend beyond China’s geographical context, enhancing the understanding of corporate overinvestment and its determinants. The practical implications of these insights are significant for companies, particularly those seeking to refine their investment strategies and governance mechanisms.

The remainder of this paper is organized as follows. Section 2 discusses the hypothesis development, and Section 3 presents the data and the methodology used. Further, Section 4 discusses the empirical results of the findings. Finally, Section 5 concludes the study.

## Literature review

### The correlation of CEO culture and firm investment

The relationship between CEO culture and firm investment has been a topic of interest among scholars due to the potential impact of cultural background on investment decision-making behavior. Studies have shown that cultural preferences for risk can vary across different backgrounds, leading to differences in investment decisions [10, 11]. In recent years, evidence from Chinese sub-national institutional contingencies also showed how tournament incentives could spur CSR performance [12]. Personal traits, such as values and risk tendencies, can also impact decision-making [13]. The upper echelon theory suggests that top management teams’ characteristics can influence a company’s strategy and performance, with the CEO being a crucial figure in determining the company’s risk decisions [14, 15]. Further studies on the effect of CEO characteristics on firm performance across different cultural contexts, such as the study of Saudi Arabia Listed Firms, supplement this perspective [16]. Understanding the relationship between CEO culture and firm investment can provide valuable insights into the role of culture in investment decision-making and potentially aid companies in improving their investment strategies. The influence of financial decisions, like capital structure, on corporate performance also plays a vital role [17].

Traditional culture significantly impacts on an individual’s background, encompassing elements such as diet, religion, history, language, and personal habits. In China, a vast land with numerous ethnic groups, the main geographic boundary is the north-south division. The formation of different cultures in southern and northern China is influenced by factors such as climate, geography, and history, with geography being the most stable and influential factor in cultural formation [18, 19]. The north and south are divided geographically and culturally by the Qinling Mountains and the Huai River line, which corresponds with the zero-degree average temperature line in January, a boundary for the survival of winter crops. In terms of agriculture, the south mainly cultivates rice, while the north predominantly cultivates wheat. In comparison to Western countries with well-developed economic systems, traditional culture in China has a more profound impact on the economy than formal institutions.

The difference between southern and northern cultures is shaped by the natural environment and production methods, with a character that is more adventurous, independent, and brave in the north and more commercially savvy, meticulous, and risk-averse in the south. Drawing on the cultural-attitude-behavior hypothesis [20] and the upper echelon theory[16], this study proposes that there is a correlation between CEO cultural background and corporate investment decisions. Specifically, we hypothesize that firms led by CEOs with northern cultural backgrounds will exhibit a greater inclination towards overinvestment compared to those led by CEOs with southern cultural backgrounds. This is due to the fact that northern culture tends to exhibit less uncertainty avoidance and greater risk preference, leading to a higher propensity for overinvestment. Therefore, the following hypothesis is proposed:

Hypothesis 1: Firms led by CEOs with northern cultural backgrounds are more likely to engage in overinvestment than those led by CEOs with southern cultural backgrounds.

### The relationship of CEO age and firms investment

The upper echelons theory proposes that top management teams’ characteristics significantly influence a company’s strategy and performance [21]. Previous study [22], has found that executive traits are crucial factors affecting corporate investment decisions. Moreover, there has been growing interest in exploring the effect of CEO personal characteristics, including age, on company decision-making [23]. Stduy has shown that CEO age can influence investment decisions and attitudes towards risk, as well as other company policies [24, 25]. Age is easily observable and has been shown to have different outcomes on corporate decisions, with studies suggesting that the average age of the top management team can affect organizational growth [26].

Previous research has demonstrated that CEO age can significantly affect corporate investment decisions, with younger CEOs tending to take greater risks and be more aggressive in their investment decisions compared to older CEOs. In contrast, older CEOs tend to adopt lower-risk investment strategies and exhibit a more conservative ddecision-making style, reducing company risk through policies such as lower financial leverage, more diversified investments, and lower investment expenditures [27, 28]. The managerial signaling model developed predicts that younger CEOs will be more aggressive in investing [7]. However, from the perspective of managerial signaling, there is a negative correlation between CEO age and risk-taking behavior, indicating that younger CEOs take on more risk compared to older managers. Prior studies found that older CEOs tend to avoid investing in high-risk projects and make more conservative decisions compared to younger CEOs [29, 30]. Older CEOs tend to seek more information, conduct more in-depth evaluations, spend more time making decisions, demonstrate greater commitment to the company, and put in more effort to achieve the company’s goals [31]. Three reasons why younger CEOs are more aggressive in investing: First, older CEOs may be in a period of life where financial security and job security are more important. Second, older CEOs may have a greater commitment to the company’s current state. Finally, the mental and physical stamina of older CEOs may be weaker, or they may be unable to grasp new ideas and learn new behaviors.

Therefore, this study, in line with the managerial signaling model [7] and the reviewed literature, hypothesizes a negative correlation between CEO age and risk-taking behavior. It predicts that older CEOs tend to adopt lower-risk investment policies to reduce the firm’s risk exposure. Accordingly, it posits that as CEO age increases, risk avoidance and conservative decision-making tendencies increase. Furthermore, it anticipates that older CEOs can better resist the influence of a Northern cultural background on excessive investment behavior. Thus, the following hypothesis is proposed:

Hypothesis 2: There is a negative correlation between CEO age and risk-taking behavior. The older the CEO, the more likely they are to adopt a conservative investment policy and resist the influence of a Northern cultural background on investment behavior.

### The correlation of firm’s investment and ownership structure in China

The ownership structure of Chinese state-owned enterprises (SOEs) has significantly influenced their investment decisions [3]. Due to the lack of proper supervision and corporate governance in SOEs, the ownership structure is often overlooked, and executives prioritize political objectives over market-oriented ones, leading to severe agency problems [12]. This issue is compounded by the lack of protection for minority shareholders in state-owned listed companies[32]. On the other hand, privately-owned or township enterprises that have gone public and become listed companies have relatively market-oriented ownership structures, resulting in stricter management supervision and fewer agency problems [6].

In Chinese state-owned enterprises, the ownership structure is highly interconnected, which leads to improper supervision and neglect of ownership. Additionally, state-owned enterprises suffer from imperfect corporate governance, with executive personnel often selected by their respective local governments, and their career transitions and promotions mainly controlled by their parent company or the state. As a result, executives of listed companies often prioritize political motivations over market-oriented ones, leading to serious agency problems [9, 17]. In China, the protection of minority shareholders in state-owned listed companies has become a serious issue, as they face significant hurdles in obtaining fair treatment and obtaining appropriate compensation in the event of mergers and acquisitions [9].

Ownership structure plays a crucial role in the investment decisions of Chinese state-owned enterprises (SOEs). Due to inadequate supervision and corporate governance in SOEs, ownership is often overlooked, and executives prioritize political goals over market-oriented ones, leading to severe agency problems. Minority shareholder protection is also a significant issue in state-owned listed companies. Conversely, privately-owned or township enterprises that have gone public and become listed companies usually have market-oriented ownership structures, resulting in stricter management supervision and fewer agency problems. In Chinese state-owned enterprises, the ownership structure is highly interconnected, and executives pursue political motivations, leading to serious agency problems. Moreover, imperfect corporate governance in state-owned enterprises results in executive personnel selected by their respective local governments, with their career transitions and promotions mainly determined by their parent company or the state.

Research indicates that state-owned holding listed companies have significantly higher agency costs than non-state-owned holding companies [33], and state-owned enterprises are more likely to engage in overinvestment due to overconfidence among management [34]. Political relationships negatively affect investment efficiency in state-owned enterprises, with investment expenditures being less sensitive to growth opportunities than those of non-state-owned enterprises [9]. State-owned enterprises also have higher corporate investment due to government intervention and lower financing constraints, increasing the likelihood of overinvestment [35]. Non-state-owned enterprises have been found to have higher investment efficiency than state-owned enterprises [36].

Therefore, based on past literature, this study posits that the ownership structure of Chinese SOEs has a significant impact on their investment decisions, with agency problems being more severe due to the lack of proper supervision and corporate governance. Compared to privately-owned or township enterprises, SOEs often prioritize political objectives, leading to overinvestment and lower investment efficiency. Hence, we hypothesize:

Hypothesis 3: State-owned enterprises are more likely to engage in overinvestment compared to privately-owned or township enterprises due to agency problems resulting from political objectives.

## Methodology

Our research methodology involves an empirical investigation using data from Chinese firms, allowing us to study the real-world impact of these factors. We employ regression analysis to understand the relationships and interactions among these variables. The findings from this study contribute to both theoretical understandings and practical applications of corporate investment decision-making, with implications for companies in China and beyond.

### Data

This study leverages data from the CSMAR Guotai’an database, encompassing annual data from 2000 to 2018 for Chinese A-share listed companies, including CEO background, control variables, and data necessary to quantify overinvestment. The period from 2000 to 2018 is selected because it marks the maturation phase of China’s modern corporate governance structures and market reforms. This period also allows sufficient temporal breadth to observe long-term patterns and trends in CEO behavior and overinvestment. Financial institutions, given their unique investment and financing behaviors and their susceptibility to stringent government and regulatory supervision, are excluded from the study to eliminate any potential distortions or sector-specific anomalies in the analysis. Furthermore, this study does not consider B shares and H shares due to their different investor base and regulation, focusing solely on A-share listed companies to ensure data consistency and reliability.

### Variables definition

#### Overinvesment

This study uses the research framework proposed [37] to calculate overinvestment. First, the total investment is calculated as the sum of the investment required to maintain current assets and the investment needed for new assets. The formula is expressed as Eq (1)

$${\text{I}}_{\text{TOTAL},\text{t}}={\text{CAPEX}}_{\text{t}}+{\text{Acquisitions}}_{\text{t}}-{\text{SalePPE}}_{\text{t}}$$ (1)

where, total investment(I_total_)is defined as the sum of capital expenditure(CAPEX)and acquisition expenditure(Acquisitions), minus the proceeds from the sale of property, plant, and equipment(SalePPE) Additionally, total investment(I_TOTAL_) can be divided into two parts: investment for maintaining assets (I_MAINTENANCE_) and investment for new projects (I_NEW_). The equations are as follows (2):

$${\text{I}}_{\text{TOTAL},\text{t}}={\text{I}}_{\text{NEW},\text{t}}+{\text{I}}_{\text{MAINTENANCE},\text{t}}$$ (2)

Thus, new project investment can be divided into two parts: expected investment with a positive net present value ($I_{NEW}^{*}$) and abnormal or unexpected investment ($I_{NEW}^{\varepsilon }$). The value of abnormal investment can be positive or negative, with the positive portion considered as overinvestment and the negative portion considered as underinvestment. Since this study focuses on overinvestment, we chose the positive portion of abnormal investment as the research target. The relationship equation is as follows::

$${\text{I}}_{\text{NEW},\text{t}}={\text{I}}_{\text{NEW},\text{t}}^{*}+{\text{I}}_{\text{NEW},\text{t}}^{\varepsilon }$$ (3)

Next, this study will use the investment expenditure formula to calculate expected investment($I_{NEW}^{*}$) and abnormal investment($I_{NEW}^{\varepsilon }$). The fitted values from Eq (4) correspond to expected investment ($I_{NEW}^{*}$), while the residual values correspond to abnormal investment ($I_{NEW}^{\varepsilon }$)The equation is as follows:

$$\begin{array}{ll} {\text{I}}_{\text{NEW},\text{t}} & =\beta_{0}+\beta_{1}{\frac{\text{V}}{\text{P}}}_{\text{t}-1}+\beta_{2}{\text{Leverage}}_{\text{t}-1}+\beta_{3}{\text{Cash}}_{\text{t}-1}+\beta_{4}{\text{Age}}_{\text{t}-1}+\beta_{5}{\text{Size}}_{\text{t}-1}+\\ & \beta_{6}{\text{Stockreturns}}_{\text{t}-1}+\beta_{7}{\text{I}}_{\text{New},\text{t}-1}+\sum \text{Yeardummy}+\sum \text{Industrydummy}+{\text{I}}_{\text{New},\text{t}}^{\varepsilon } \end{array}$$ (4)

To measure V/P company growth, the calculation method is to divide the value of operating assets (V_AIP_) by the equity market value, and the calculation formula for operating asset value V_AIP_ = (1 − αr) BV + α(1 + r) X-αrd is based on the abnormal earnings persistence parameter estimated from the Ohlson (1995) research framework. The calculation method of α is, α = (ω/(1 + r − ω)) for operating asset value is based on the abnormal earnings persistence parameter estimated from the research framework [38]; r is the discount rate used in [27] "r" is estimated to be 0.05 according to Chen et al. (2016), and "ω" is estimated to be 0.62 according to Ohlson (1995), and ω is estimated to be 0.62 [39]. BV is the book value of common stock, X is earnings after deducting depreciation expense, and d is dividends. is total liabilities divided by total assets, is short-term investments divided by total assets, is the natural logarithm of the year in which the company went public, is the natural logarithm of total assets, is the difference in market value between this year and last year, is a dummy variable controlling for calendar year fixed effects, and is a dummy variable controlling for fixed effects across different industries.

### Independent variables

#### CEO cultural background

The CEO cultural background is a dummy variable. This study defines CEO cultural background based on the "New Geographical Literature" in 1908 that the Qinling Mountains-Huaihe River line serves as the geographical north-south boundary in China. If the birthplace of the CEO of company i in year t belongs to a northern province, Culture = 1. If it belongs to a southern province, Culture = 0.

#### CEO age

CEO age data obtained.from the CSMAR database, This study defines CEO age as the age of the CEO of the i-th company in the t-th year [28].

#### State-owned Enterprises (SOE)

The relevant information of listed companies will be obtained from the CSMAR database. If the company is a state-owned enterprise, then SOE = 1, and if it is a non-state-owned enterprise, then SOE = 0.

### Control variables

Based on past literature, this study will control for certain company characteristics that may affect overinvestment, including free cash flow (FCF), leverage, company size, and firm age [28, 37]. This study defines company size as the natural logarithm of total assets, and leverage as total liabilities divided by total assets. Free cash flow is defined as operating cash flow minus expected new investments [37], and is calculated using Eqs (1), (2) and (3) as per Richard’s research framework. Operating cash flow is the cash flow generated by a company’s operations, and is calculated by subtracting cash flows related to investments in maintaining assets from cash flows generated by operations, and then adding research and development (R&D) expenses. Expected new investments are calculated using depreciation and amortization expenses obtained from financial reports. In addition, this study includes corporate governance variables as control variables, including the average shareholding percentage of the top three shareholders (Herfi3), whether the largest shareholder holds more than 25% of the shares (D1), CEO duality (Isduality), board size (BoardSize), supervisor size (SupervisorSize), executive size (ExecutiveSize), outside directors (OutsideDirectors), non-paid directors (NonpaidDirectors), and non-paid supervisors (NonpaidSupervisor). Herfi3 is defined as the average shareholding percentage of the top three shareholders, D1 is defined as 1 when the largest shareholder holds more than 25% of the shares, and 0 otherwise. Isduality is defined as 1 when the chairman and CEO are the same person, and 0 otherwise. BoardSize is defined as the total number of directors on the board, SupervisorSize is defined as the total number of directors on the supervisory board, ExecutiveSize is defined as the total number of executives on the board, OutsideDirectors is defined as the proportion of outside directors to the total number of directors on the board, NonpaidDirectors is defined as the proportion of directors who do not receive compensation from the company to the total number of directors on the board, and NonpaidSupervisor is defined as the proportion of supervisors who do not receive compensation from the company to the total number of supervisors in the company. Table 1 provides detailed definitions of these variables.

**Table 1:** Descriptive statistics.

Variables	mean	sd	p25	p50	p75	min	max
OI	0.049	0.066	0.011	0.029	0.065	0.000	3.610
Culture	0.102	0.302	0.000	0.000	0.000	0.000	1.000
CEO Age	48.015	6.567	43.000	48.000	52.000	24.000	80.000
FCF	0.009	1.144	-0.019	0.019	0.060	-0.236	1.127
Leverage	0.589	6.324	0.349	0.504	0.646	0.007	0.944
Size	21.954	1.370	21.043	21.827	22.730	12.314	28.509
Firm age	2.104	0.741	1.792	2.303	2.639	0.000	3.258
Herfi3	0.171	0.126	0.072	0.137	0.243	0.000	0.810
D1	0.279	0.449	0.000	0.000	1.000	0.000	1.000
Isduality	0.170	0.376	0.000	0.000	0.000	0.000	1.000
BoardSize	9.109	1.906	8.000	9.000	9.000	3.000	19.000
SupervisorSize	3.917	1.296	3.000	3.000	5.000	1.000	15.000
ExecutiveSize	6.396	2.497	5.000	6.000	8.000	1.000	45.000
OutsideDirectors	3.273	0.695	3.000	3.000	4.000	0.000	8.000
NonpaidDirectors	2.366	2.002	1.000	2.000	4.000	0.000	12.000
NonpaidSupervisor	1.418	1.295	0.000	1.000	2.000	0.000	11.000
N	325062						

OI refers to overinvestment, Culture refers to the CEO’s cultural background, CEO Age refers to the age of the CEO, FCF refers to free cash flow, Leverage refers to debt, Size refers to company size, Firm Age refers to the logarithm of company age, Herfi3 refers to the average shareholding ratio of the top three shareholders, D1 refers to the largest shareholder’s shareholding exceeding 25%, Isduality refers to CEO duality, BoardSize refers to the size of the board of directors, SupervisorSize refers to the size of the board of supervisors, ExecutiveSize refers to the size of the executive team, OutsideDirectors refers to external directors, NonpaidDirectors refers to non-paid directors, NonpaidSupervisor refers to non-paid

### Model development

#### The relationship between overinvestment and CEO cultural background

To test hypothesis 1: CEO with a northern cultural background will increase overinvestment in the company since CEO cultural background and corporate overinvestment are not causally related, CEO cultural background will not change due to corporate overinvestment. Therefore, there is no endogeneity issue between CEO cultural background and corporate overinvestment in this study. It is hypothesized that the two variables have a positive correlation (>0), and therefore, this study establishes the following regression model for estimation

$${\text{OI}}_{\text{i},\text{t}}=\alpha_{0}+\alpha_{1}{\text{Culture}}_{\text{i},\text{t}}+\theta_{1}{\text{CV}}_{\text{i},\text{t}}+\varepsilon_{\text{i},\text{t}}$$ (5)

In this study, the variable for overinvestment is represented by "I", and the variable for CEO’s cultural background is represented by "Culture". When the CEO’s birthplace is in the northern region, it is considered to have a northern cultural background, and Culture is assigned a value of 1. If the birthplace is in the southern region, Culture is assigned a value of 0. The study also includes 14 control variables: free cash flow, debt, company size, company age, average shareholding ratio of top three shareholders, maximum shareholder holding more than 25%, CEO duality, board size, supervisor size, executive size, external directors, non-remunerated directors, non-remunerated supervisors, and fixed effects for year and industry.

#### CEO age moderate the effect of CEO cultural background on overinvestment

To test hypothesis two: whether CEO age moderates the relationship between overinvestment and CEO cultural background, we hypothesize a negative correlation between the two variables, α_3_ < 0. Therefore, we establish the following regression model for estimation in this study:

$${\text{OI}}_{\text{i},\text{t}}=\alpha_{0}+\alpha_{1}{\text{Culture}}_{\text{i},\text{t}}+\alpha_{2}\text{CEO}\;{\text{Age}}_{\text{i},\text{t}}+\alpha_{3}{\text{Culture}}_{\text{i},\text{t}}\times CEO\;Age_{i,t}+\theta_{1}{\text{CV}}_{\text{i},\text{t}}+\varepsilon_{\text{i},\text{t}}$$ (6)

The variables in the model are as follows: Overinvestment (a binary variable indicating whether the firm is overinvesting), Culture (a binary variable indicating whether the CEO has a northern cultural background, with 1 for a northern birthplace and 0 for a southern birthplace), Age (the age of the CEO), and 14 control variables including Free Cash Flow, Debt, Company Size, Company Age, Average Shareholding Ratio of the Top Three Shareholders, Maximum Shareholding of a Single Shareholder (>25%), CEO Duality, Board Size, Supervisor Size, Executive Size, External Directors, Unpaid Directors, Unpaid Supervisors, Year Fixed Effects, and Industry Fixed Effects.

#### Overinvestment and the impact of CEO cultural background

To test hypothesis three: Holding other conditions constant, compared to non-state-owned enterprises, the age of state-owned enterprise CEOs will more significantly inhibit the impact of CEO’s northern cultural background on excessive investment in enterprises. It is estimated that the two are negatively correlated, α_3_ and β_3_ < 0. Therefore, this study establishes the following regression model:

$$\begin{array}{ll} {\text{OI}}_{\text{i},\text{t}} & =\left(\alpha_{0}+\alpha_{1}{\text{Culture}}_{\text{i},\text{t}}+\alpha_{2}{\text{Age}}_{\text{i},\text{t}}+\alpha_{3}{\text{Culture}}_{\text{i},\text{t}}\times Age_{i,t}\right)\times {\text{NONSOE}}_{i,t}\\ & +\theta_{1}{\text{CV}}_{\text{i},\text{t}}\times {\text{NONSOE}}_{i,t}+\varepsilon_{\text{i},\text{t}} \end{array}$$ (7)

$$\begin{array}{ll} {\text{OI}}_{\text{i},\text{t}} & =\left(\beta_{0}+\beta_{1}{\text{Culture}}_{\text{i},\text{t}}+\beta_{2}{\text{Age}}_{\text{i},\text{t}}+\beta_{3}{\text{Culture}}_{\text{i},\text{t}}\times Age_{i,t}\right)\times {\text{SOE}}_{i,t}\\ & +\theta_{2}{\text{CV}}_{\text{i},\text{t}}\times {\text{SOE}}_{i,t}+\varepsilon_{\text{i},\text{t}} \end{array}$$ (8)

In this study, overinvestment serves as the dependent variable, while the CEO’s cultural background is the primary independent variable of interest. CEOs born in the North are indicated by a value of 1, while those born in the South are assigned a value of 0. The control variables include non-state-owned and state-owned enterprises, as well as 14 additional variables: free cash flow, debt, company size, company age, average shareholding ratio of the top three shareholders, maximum shareholder holding > 25%, CEO duality, board size, supervisor size, executive size, external directors, unpaid directors, unpaid supervisors, and fixed effects for year and industry.

To test the hypothesis, this study employs Chow’s test, which assesses whether the linear regression coefficients of two distinct datasets are equal. Widely utilized in time series analysis, Chow’s test detects the presence of structural changes and was developed by economist Gregory Chow in 1960. The method involves estimating the same variable across two different periods or samples to identify any disparities.

In this context, the samples are divided into state-owned enterprises and non-state-owned enterprises. Chow’s test examines whether a significant difference exists in the coefficients between Eqs (6) and (7), assuming constant residuals. The null hypothesis is H0: β1 = β2 = 0. If a difference is detected, the null hypothesis is rejected, signifying a divergence between the two groups. Consequently, the study separates the sample into state-owned and non-state-owned enterprises, applying Chow’s test to evaluate the hypothesis.

### 1. Empirical result

#### Descriptive statistic

**Fig 1:**
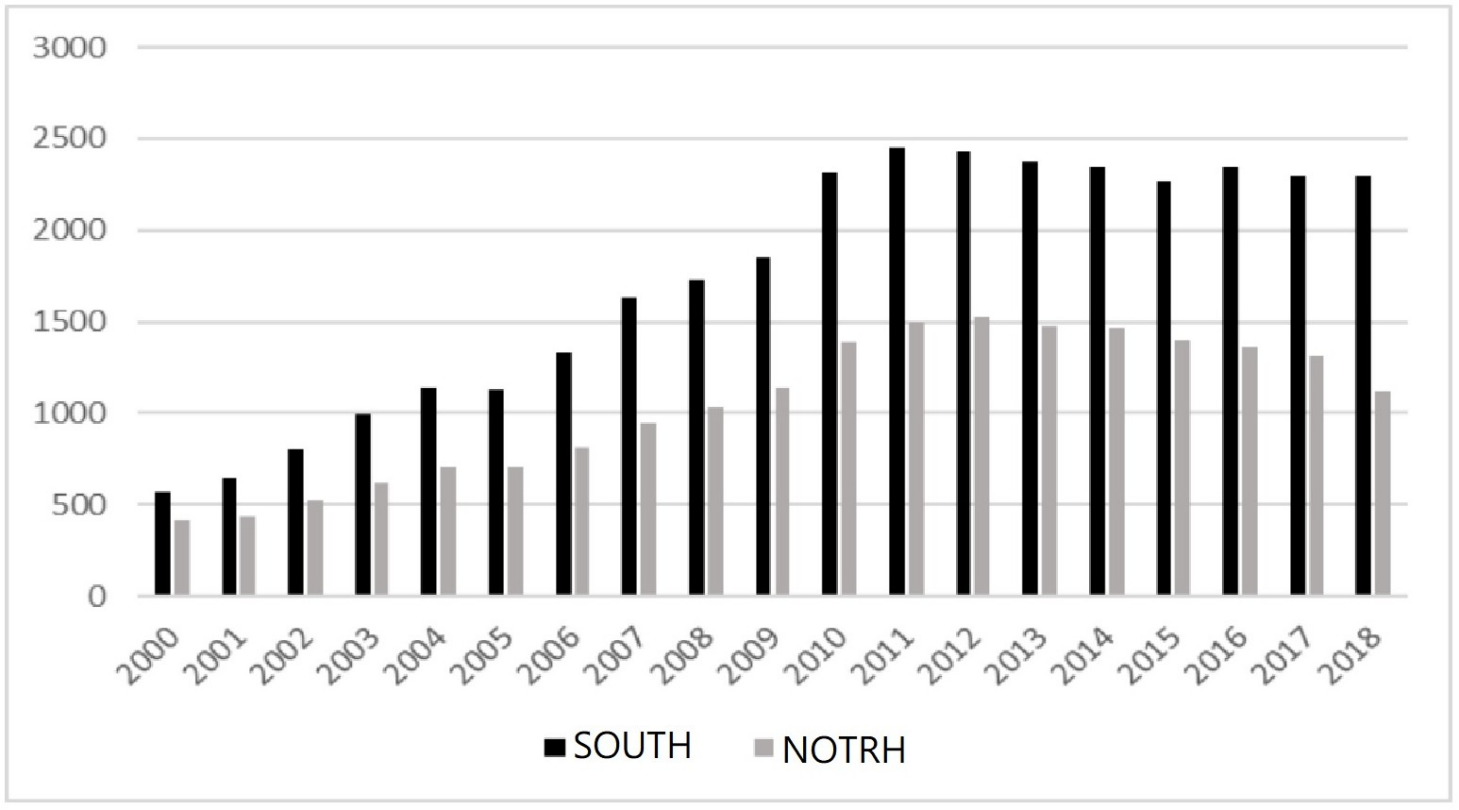
Distribution chart of CEO cultural backgrounds.

In Table 1, during the sample period, the average level of overinvestment in non-financial industries in China was 0.049, with a standard deviation of 0.066. The difference between the maximum value (3.610) and the minimum value (0.000) is 3.610, indicating that there is a large fluctuation in overinvestment by Chinese firms, which can lead to overinvestment or underinvestment under different corporate governance and business conditions. The average CEO cultural background is 0.102 with a standard deviation of 0.302. It can be observed that a larger number of CEOs are from southern China (Fig 1 Distribution chart of CEO cultural backgrounds). The average CEO age is 48.015 years, with a difference of 56 years between the maximum value (80.00) and the minimum value (24.00). The average free cash flow is 0.009, indicating that Chinese companies generally have inadequate control over cash flow, resulting in insufficient cash flow. The difference between the maximum value (1.127) and the minimum value (-0.236) is 1.363, indicating that there is a significant difference in the free cash flow held by companies with different operating conditions.

Other variables include the average and standard deviation of total liabilities, which are 0.589 and 6.324, respectively. The average company size is 21.954, with a median of 21.827. The average company age is 2.104. Corporate governance variables include the average and standard deviation of the top three shareholders’ ownership ratio, which are 0.171 and 0.126, respectively. The average ownership percentage of the largest shareholder is 0.279. The average CEO duality is 0.170. The average size and standard deviation of the board of directors are 9.109 and 1.906, respectively, while the average size and standard deviation of the board of supervisors are 3.917 and 1.296, respectively. The average size and standard deviation of the senior management team are 6.396 and 2.497, respectively. The average and standard deviation of external directors are 3.273 and 0.695, respectively, while the average and standard deviation of non-compensated directors are 2.366 and 2.002, respectively, and the average and standard deviation of non-compensated supervisors are 1.418 and 1.295, respectively. From a financial perspective, the article discusses the phenomenon of overinvestment among non-financial firms in China. It notes that the average level of overinvestment among these firms during the sample period was 0.049, with a standard deviation of 0.066. This indicates that there is a high degree of volatility in overinvestment levels among Chinese firms, which can lead to both overinvestment and underinvestment under different corporate governance and operational conditions.

### Correlation of cultural background and firms’ overinvesmt

To investigate the relationship between CEO cultural background and overinvestment, this study used a least squares regression model for empirical analysis, and the results are shown in Table 2. Column 1 of Table 3 shows that at the 1% significance level, the coefficient of CEOs with a northern cultural background is 0.00352, indicating a significant positive correlation between overinvestment and CEOs with a northern cultural background. This is consistent with the expected hypothesis, indicating that when the CEO has a northern cultural background, they may prefer risk, which leads to an increase in overinvestment. Column 3 of Table 2 adds control variables and fixed effects. At the 1% significance level, the coefficient of CEOs with a northern cultural background is 0.00337, which is consistent with the expected hypothesis, indicating a significant positive correlation between overinvestment and CEOs with a northern cultural background. This explains "culture-attitude-behavior" hypothesis [39]. Compared with southern Chinese culture, northern Chinese culture has a greater risk preference. Therefore, this study believes that when the CEO has a northern cultural background, overinvestment is more likely to occur.

**Table 2:** Regression of CEO cultural background and firms’ overinvestment.

	(1)	(2)	(3)
	OI	OI	OI
Culture	0.0792**	0.3860***	0.3374***
	(2.09)	(7.46)	(7.57)
fcfta2	1.7499**	1.7342*	1.7345*
	(1.96)	(1.95)	(1.95)
leverage	0.0098***	0.0102***	0.0097***
	(4.25)	(3.97)	(3.80)
size	0.5552***	0.7047***	0.7433***
	(6.80)	(7.38)	(6.77)
lagea	-1.0780***	-1.4542***	-1.5274***
	(-21.40)	(-20.47)	(-22.53)
d1		-1.0020***	-1.1730***
		(-3.02)	(-3.37)
d2		-0.9241***	-1.0562***
		(-2.59)	(-2.73)
isduality		-1.2180***	-1.1985***
		(-5.78)	(-5.68)
herfi3		-4.9027***	-5.1275***
		(-27.53)	(-29.01)
BoardSize		-0.3100***	-0.3198***
		(-2.85)	(-2.96)
SupervisorSize		-0.2240***	-0.2333***
		(-7.50)	(-6.48)
ExecutiveSize		-0.0079	0.0016
		(-0.62)	(0.10)
OutsideDirectors		1.0226***	1.0179***
		(3.28)	(3.32)
NonpaidDirectors		-0.0427	-0.0489
		(-1.19)	(-1.38)
Industry Effect	No	No	Yes
Year Effect	No	No	Yes
N	376424	325110	325110
Adj-*R*^2^	0.0120	0.0144	0.0147

OI refers to overinvestment, Culture refers to CEO cultural background, CEO Age refers to CEO age, FCF refers to free cash flow, Leverage refers to debt, Size refers to company size, Firm Age refers to company age in natural logarithm, Herfi3 refers to the average shareholding ratio of the top three shareholders, D1 refers to the largest shareholder holding>25%, Isduality refers to CEO duality, BoardSize refers to board size, SupervisorSize refers to supervisor size, ExecutiveSize refers to executive size, OutsideDirectors refers to outside directors, NonpaidDirectors refers to non-paid directors, NonpaidSupervisor refers to non-paid supervisors, and Industry & Year Effect refers to fixed effects of industry and calendar year. Numbers in parentheses are t-statistics.

*, **, *** denote statistical significance at the 10%, 5%, and 1% level, respectively.

**Table 3:** Regression results for the relationship between CEO cultural background, overinvestment, and CEO age.

	(1)	(2)	(3)
	OI	OI	OI
Culture	2.0498***	3.2179***	3.0675***
	(7.80)	(10.88)	(10.28)
CEO Age	-0.0149***	-0.0188***	-0.0208***
	(-6.22)	(-4.18)	(-3.76)
Culture×CEO Age	-0.0346***	-0.0577***	-0.0556***
	(-6.59)	(-9.75)	(-9.32)
Control Variables	No	Yes	Yes
Industry Effect	No	No	Yes
Year Effect	No	No	Yes
N	376200	325062	325062
Adj-*R*^2^	0.0000711	0.0144	0.0147

OI stands for overinvestment, Culture refers to the CEO’s cultural background, CEO Age refers to the CEO’s age, and Control Variables include free cash flow, leverage, firm size, firm age, Herfi3 (average ownership percentage of the top three shareholders), D1 (ownership percentage of the largest shareholder exceeding 25%), Isduality (whether the CEO holds the position of chairman and CEO), BoardSize (board size), SupervisorSize (supervisor size), ExecutiveSize (executive size), OutsideDirectors (number of outside directors), NonpaidDirectors (number of non-paid directors), NonpaidSupervisor (number of non-paid supervisors). Industry & Year Effect represents the fixed effects of industry and calendar year. *Numbers in parentheses are t-statistics.

*, **, *** denote statistical significance at the 10%, 5%, and 1% level, respectively.

This study finds that over-investing is more sensitive to current free cash flow and consistent with the explanation of agency costs, as there is a positive correlation between over-investing and higher levels of free cash flow. The results indicate that companies with positive free cash flow are more likely to over-invest, which is consistent with prior literature [41–43]. Among other corporate governance variables, this study finds that larger supervisor size can better suppress over-investing, which is consistent with [28]. At the 1% significance level, the coefficient is -0.00320, indicating a negative correlation. It is found that both the average shareholding of the top three shareholders (Herfi3) and the largest shareholder holding >25% (D1) are negatively related to over-investing, with coefficients of -0.0513 and -0.0117, respectively, indicating that more concentrated ownership can better suppress over-investing. The occurrence of over-investing significantly decreases as company age increases, and both are negatively correlated. The opportunity for over-investing increases with company size, and both are positively correlated.

Flow, which is consistent with the explanation of agency costs. When free cash flow is high, overinvestment by firms tends to be positively correlated with it. This indicates that companies with positive free cash flow are more likely to overinvest [40–42]. Among other corporate governance variables, this study found that larger board size can better restrain firms from overinvestment, which is consistent with [28]. At the 1% significant level, the two variables show a negative correlation with a coefficient of -0.00320. It can also be found that the average shareholding of the top three shareholders (Herfi3) and the largest shareholder’s shareholding >25% (D1) are both negatively correlated at the 1% significant level, with coefficients of -0.0513 and -0.0117, respectively. This indicates that the more concentrated ownership is, the better it can restrain firms from overinvesting. When a firm’s age increases, the likelihood of overinvestment decreases, and both variables show a negative correlation. When a firm’s size increases, the likelihood of overinvestment also increases, and both variables show a positive correlation.

### The correlation of CEO age among CEO cultural background, firms’ overinvestment

To examine the relationship between CEO cultural background, overinvestment, and CEO age, this study includes CEO age as a control variable. In Table 2, column 1, without control variables and at a 5% significance level, the coefficient of CEOs with a northern cultural background is 0.0205, indicating a significant positive correlation between overinvestment and CEOs with a northern cultural background. After including CEO age as a control variable, the two variables show a negative correlation at a 5% significance level, with a coefficient of -0.000346. This means that as CEO age increases, it is more effective in suppressing the relationship between CEOs with a northern cultural background and overinvestment [7, 8, 28].

In Table 3, column 3, with control variables and at a 1% significance level, the coefficient of CEOs with a northern cultural background is 0.00307, indicating a significant positive correlation between overinvestment and CEOs with a northern cultural background. This shows that when CEOs have a northern cultural background, it can lead to an increase in overinvestment in the company. Regarding CEO age, it is found that CEO age suppresses the impact of overinvestment. At a 1% significance level, the coefficient is -0.000208, indicating a negative correlation between CEO age and overinvestment. This means that as CEO age increases, it is more effective in suppressing overinvestment in the company [7, 8, 28]. When multiplying CEO cultural background and CEO age, at a 1% significance level, the coefficient is -0.000556, indicating that CEO age can suppress the impact of CEOs with a northern cultural background on overinvestment. Therefore, this supports Hypothesis 2, indicating that as CEO age increases, it is more effective in suppressing the relationship between CEOs with a northern cultural background and overinvestment.

This study finds that CEO age can effectively suppress overinvestment in the company, which is consistent with the manager signaling model. Therefore, this study concludes that as CEO age increases, it is more effective in suppressing the relationship between CEOs with a northern cultural background and overinvestment.

### Relationship between CEO cultural background, overinvestment, and CEO age grouped by ownership structure

Due to agency problems, companies may experience overinvestment. In China, state-owned enterprises are the backbone of the economy. Previous research has shown a relationship between state-owned enterprises and overinvestment, which is believed to be caused by excessive confidence in management. State-owned enterprises in China have more severe agency problems and lower monitoring effectiveness, which makes them more prone to overinvestment. Therefore, this study divided the sample into state-owned and non-state-owned enterprises to investigate the differences between the two.

Table 4 distinguishes between state-owned and non-state-owned enterprises. At a 1% significance level, it was found that in state-owned enterprises, CEOs with a northern cultural background are positively correlated with overinvestment, with a coefficient of 0.0499, while in non-state-owned enterprises, the coefficient is not significant, at -0.00464. After adding CEO age, at a 1% significance level, it was found that CEO age can effectively suppress the positive correlation between CEO’s northern cultural background and overinvestment in state-owned enterprises, with a negative correlation coefficient of -0.000955, while in non-state-owned enterprises, it is not significant, with a coefficient of 0.0000686. This shows that the effects of CEO cultural background, overinvestment, and CEO age are more significant in state-owned enterprises. Therefore, this supports Hypothesis 3, indicating that the effect is more significant for state-owned enterprises than for non-state-owned enterprises.

**Table 4:** The effect of equity structure segmentation.

	Non-state-owned enterprises	State-owned enterprises
	(1)	(2)
	OI	OI
Culture	-0.4617	4.9920***
	(-0.92)	(9.26)
CEO Age	-0.0784***	-0.0056
	(-14.35)	(-0.70)
Culture×CEO Age	0.0068	-0.0956***
	(0.68)	(-9.66)
Control Variables	Yes	Yes
Industry Effect	Yes	Yes
Year Effect	Yes	Yes
N	191724	133338
Adj-*R*^2^	0.589	0.0929
Chow test	Culture* CEO Age	21.51***
	Culture	23.78***

OI refers to overinvestment, Culture refers to CEO cultural background, CEO Age refers to CEO age, Control Variables include free cash flow, debt, company size, company age, average shareholding percentage of the top three shareholders, maximum shareholder holding over 25%, CEO duality, board size, supervisor board size, executive team size, external directors, non-remunerated directors, non-remunerated supervisors, Industry & Year Effect refers to fixed effects of industry and calendar year. Numbers in parentheses are t-statistics.

*, **, *** denote statistical significance at the 10%, 5%, and 1% level, respectively.

At the bottom of Table 4, this study used the Chow test. This test estimates whether two samples have differences based on the same sample data in two different periods or different samples. It can be seen that when the sample is divided into state-owned and non-state-owned enterprises, at a 1% significance level, the coefficient is 21.51, indicating that there is a difference between the coefficients of the two.

### Robustness

To further expand this study, geographic clustering, period clustering, Tobit model, and alternative variables were used for robustness tests. Geographic clustering was done by dividing companies into North and South based on their registered addresses, with the Qinling-Huaihe line as the boundary between North and South China. Period clustering was done by dividing years into recession periods (2007, 2008, and 2015) and non-recession periods (other years). Alternative variables were used to construct an expected investment model, as a substitute variable for overinvestment [43].

#### The effect of geographic location grouping on the company

In Table 5, this study divides the sample into southern and northern companies based on their geographical location. At the 1% significance level, it can be observed that both CEO cultural background and overinvestment are positively and significantly correlated, regardless of whether the company is located in the south or north. When the company is located in the south, the coefficient for the influence of a CEO with a northern cultural background is larger at 0.0383, compared to 0.0248 when the company is located in the north. This suggests that when a CEO with a northern cultural background is in charge of a southern cultural company, they would bring in northern culture and influence the company’s investment decision, which is consistent with the upper echelon theory that emphasizes the impact of top management team characteristics on a company’s strategy and performance. When discussing CEO age, it is found that CEO age suppresses the relationship between CEO cultural background and overinvestment to a greater extent in southern companies, with a coefficient of -0.000727, compared to -0.000365 in northern companies. At the bottom of Table 5, the CHOW test is used, which estimates whether there is a difference between two identical samples in different time periods or under different sample data. It is found that there is a significant difference in both CEO cultural background and the interaction between CEO cultural background and age after they are grouped.

**Table 5:** Results of geographical location grouping of companies.

	South firms	North firms
	(1)	(2)
	OI	OI
Culture	3.8344***	2.1901***
	(5.86)	(5.97)
CEO Age	0.0023	-0.0470***
	(0.58)	(-4.64)
Culture×CEO Age	-0.0727***	-0.0325***
	(-5.46)	(-4.27)
Control Variables	Yes	Yes
Industry Effect	Yes	Yes
Year Effect	Yes	Yes
N	136157	188905
Adj-*R*^2^	0.0190	0.0172
CHOW test	Culture*CEO Age	17.38***
	Culture	18.15***

OI refers to overinvestment, Culture refers to CEO cultural background, CEO Age refers to CEO age, Control Variables include free cash flow, debt, company size, company age, average shareholding ratio of top three shareholders, the largest shareholder’s shareholding >25%, CEO duality, board size, supervisor size, executive size, outside directors, non-paid directors, non-paid supervisors, and Industry & Year Effect represents industry and calendar year fixed effects. Numbers in parentheses are t-statistics.

*, **, *** denote statistical significance at the 10%, 5%, and 1% level, respectively.

#### Effectiveness of time period segmentation

In Table 6, the sample is divided into recession and non-recession periods, with 2007, 2008, and 2015 being the recession periods, while the other years are non-recession periods. In Table 6, it can be found that the effect is significant in both the recession and non-recession periods, consistent with the results in Table 3. In column 1 of Table 6, at a 1% significance level, the CEO’s cultural background and the overinvestment of the company are positively correlated during the recession period, with a coefficient of 0.0914. In column 2, at a 1% significance level, the effect is positively correlated, with a coefficient of 0.0295. Regardless of the recession or non-recession periods, CEO age can effectively suppress the relationship between CEO cultural background and overinvestment of the company, indicating that overinvestment is more likely to occur in companies during a recession. When the CEO is older, they can better suppress the relationship between CEO cultural background and overinvestment of the company.

**Table 6:** Effectiveness of time period segmentation.

	Recession period	Non-recession period
	(1)	(2)
	OI	OI
Culture	9.1440***	2.9500***
	(5.96)	(9.64)
CEO Age	0.0040	-0.0233***
	(0.38)	(-4.01)
Culture×CEO Age	-0.1634***	-0.0534***
	(-5.15)	(-8.78)
Control Variables	Yes	Yes
Industry Effect	Yes	Yes
Year Effect	Yes	Yes
N	18542	306520
Adj-*R*^2^	0.521	0.0164

OI refers to overinvestment, Culture refers to CEO cultural background, CEO Age refers to CEO age, Control Variables include free cash flow, leverage, firm size, firm age, average shareholding ratio of top three shareholders, largest shareholder holding >25%, CEO duality, board size, supervisor size, executive size, outside directors, non-paid directors, non-paid supervisors, and Industry & Year Effect are fixed effects for industry and calendar year. Numbers in parentheses are t-statistics.

*, **, *** denote statistical significance at the 10%, 5%, and 1% level, respectively.

#### Tobit model effect

In this study, the Tobit model is used (note), and in column 2 of Table 7, it can be seen that the coefficient of CEO with a northern cultural background is 0.0457 at a 1% level of significance, indicating a significant positive correlation between the influence of CEO with a northern cultural background and corporate overinvestment, which is consistent with the hypothesis, suggesting that when a company’s CEO has a northern cultural background, they are more likely to prefer risk, resulting in an increase in overinvestment. Multiplying CEO cultural background and age, the coefficient is -0.000838 at a 1% level of significance, indicating that CEO age can inhibit the influence of CEO with a northern cultural background on corporate overinvestment, which is consistent with hypothesis 2, suggesting that the older the CEO, the more they can suppress the relationship between CEO with a northern cultural background and corporate overinvestment. These results are consistent with those in Table 3.

**Table 7:** Tobit.

	(1)	(2)
	OI	OI
Culture	4.4178***	4.5730***
	(8.79)	(9.09)
CEO Age	-0.0215***	-0.0176***
	(-6.46)	(-5.28)
Culture×CEOAge	-0.0818***	-0.0838***
	(-8.00)	(-8.20)
Control Variables	Yes	Yes
Industry Effect	NO	Yes
Year Effect	NO	Yes

OI refers to overinvestment, Culture refers to CEO cultural background, CEO Age refers to CEO age, Control Variables include free cash flow, leverage, firm size, firm age, average shareholding ratio of top three shareholders, largest shareholder holding >25%, CEO duality, board size, supervisor size, executive size, outside directors, non-paid directors, non-paid supervisors, and Industry & Year Effect are fixed effects for industry and calendar year. Numbers in parentheses are t-statistics.

*, **, *** denote statistical significance at the 10%, 5%, and 1% level, respectively.

#### Alternative variables

So, we use the framework to construct the expected investment model [43]. Non-discretionary investment is measured as the deviation between actual investment and expected investment given the company’s investment opportunities (measured by sales growth rate). The relationship is expressed as follows:

$$Investment_{i,t+1}=\;\beta_{0}+\beta_{1}Sales\;Growth_{i,t}+\varepsilon_{i,t+1}$$ (9)

This study defines corporate investment as the sum of capital expenditures (Investment) and acquisition expenditures (Acquisitions), minus the resale income of sold properties, plants, and equipment (SalePPE). The sales growth rate(*Sales Growth*) is defined as the growth rate of sales between the current and the previous period, and using Eq (8), the residual value (*ε*) is considered as the non-expected investment of the company. The non-expected investment (*ε*) is divided into four quartiles, with the lowest being under-investing and the highest being over-investing. Therefore, this study uses over-investing as a substitute variable in this context.

In Table 8, column 2, at the 1% significance level, the coefficient of CEOs with a northern cultural background is 1.367, indicating a significant positive correlation between CEOs with a northern cultural background and over-investment in the company, consistent with Hypothesis 1. This study believes that in comparison to southern culture in China, the risk preference of northern culture is higher, so when the CEO of a company has a northern cultural background, it is more likely to lead to over-investment. After discussing the effect of CEO age, it can be seen that CEO age suppresses the impact of over-investment in the company. At the 1% significance level, the coefficient is -6.396, indicating a negative correlation between CEO age and over-investment in the company, indicating that the older the CEO is, the more effective it is to suppress over-investment in the company. When CEO cultural background and CEO age are multiplied together, the coefficient is -3.039 at the 1% significance level. It can be found that CEO age can effectively suppress the impact of CEOs with a northern cultural background on over-investment in the company, consistent with Hypothesis 2. This indicates that as the CEO of a company grows older, they can more effectively suppress the impact of a northern cultural background on over-investment in the company.

**Table 8:** Regression model results using alternative variables CEO cultural background, over-investment, and CEO age.

	(1)	(2)
	Over-investing	Over-investing
Culture	1.2265***	1.3671***
	(13.84)	(14.96)
CEO Age	-5.2823***	-6.3968***
	(-6.55)	(-8.24)
Culture×CEO Age	-2.5723***	-3.0394***
	(-14.74)	(-16.23)
Control Variables	Yes	Yes
Industry Effect	NO	Yes
Year Effect	NO	Yes
Adj-*R*^2^	0.252	0.287

Over-investing refers to excessive investment [42]. Culture denotes CEO’s cultural background, while CEO Age refers to CEO’s age. Control variables include free cash flow, leverage, firm size, firm age, Herfindahl index of top three shareholders, percentage of shares held by the largest shareholder exceeding 25%, CEO duality, board size, supervisor size, executive size, outside directors, non-paid directors, non-paid supervisors. Industry & Year Effect are fixed effects of industry and calendar year. Numbers in parentheses are t-statistics.

*, **, *** denote statistical significance at the 10%, 5%, and 1% level, respectively.

## Conclusion

Our research indicates a notable positive correlation between a CEO’s northern cultural background and corporate overinvestment within the context of China’s A-share listed companies from 2000 to 2018. This relationship can be attributed to the North’s distinct cultural characteristics, such as a reduced aversion to uncertainty and a higher preference for risk. We also examined the moderating effects of CEO age and company ownership on these dynamics.

Consistent with established research [7, 8, 17], we found that older CEOs tend to employ more conservative strategies. This tendency reduces the incidence of overinvestment commonly associated with a northern cultural background. Furthermore, state-owned companies showed a pronounced influence of a CEO’s northern cultural background on corporate overinvestment. This observation aligns with recent studies suggesting state-owned enterprises have elevated agency costs and managerial overconfidence [6, 9, 12].

Our study illuminates the complex role of a CEO’s cultural background, age, and the company’s ownership structure in influencing corporate overinvestment. It underscores the importance of these variables in shaping corporate governance and strategic investment decisions. Our findings add to the existing literature, providing valuable insights for researchers, investors, and policymakers. This is particularly beneficial in understanding the investment decision-making dynamics within fast-growing economies like China. However, our study also reveals the need for further exploration in this field. Future research could delve deeper into these relationships, considering other potential moderating variables and different cultural contexts. Longitudinal studies would also be beneficial in capturing these dynamics’ evolution over time.

Importantly, our findings related to Chinese state-owned enterprises serve as a foundation for comparative studies in various cultural and institutional settings. This would help discern the universal and context-specific aspects of CEO decision-making. A deeper examination of the intersection between CEO age and gender can also contribute to a more comprehensive understanding of gender disparities in decision-making. The effects of leadership transitions, particularly across generations, on investment decisions warrant more investigation, as they could yield crucial insights into shifts in investment strategies and their impacts on firm performance.

Moreover, widening the scope to include other CEO characteristics, such as educational background or personality traits, could enrich our understanding of leadership and strategic decision interplay. Additionally, the influence of board characteristics on CEO decisions, an often overlooked aspect, deserves consideration for its crucial role in guiding a firm’s trajectory. Undoubtedly, exploring these areas will significantly enrich our understanding of the intricate relationship between CEO characteristics and firm investment strategies.
